# Electronic Medical Record Tobacco Use Vital Sign

**DOI:** 10.1186/1617-9625-2-2-109

**Published:** 2004-06-15

**Authors:** John W Norris, Smita Namboodiri, Syed Haque, David J Murphy, Frank Sonneberg

**Affiliations:** 1 Division of Medical Informatics, Department of Medicine, New Jersey Medical School; 2 Department of Health Informatics, School of Health Related Professions; 3 Department of Medicine, Robert Wood Johnson Medical School; 4 University of Medicine and Dentistry of New Jersey, Newark, New Jersey

## Abstract

**Objective:**

Determination of the prevalence of tobacco use and impact of tobacco prevention/treatment efforts in an electronic medical record enabled practice utilizing a defined tobacco vital sign variable.

**Design and Measurements:**

Retrospective cohort study utilizing patient data recorded in an electronic medical record database between July 15, 2001, and May 31, 2003. Patient-reported tobacco use status was obtained for each of 6,771 patients during the pre-provider period of their 24,824 visits during the study period with the recorder blinded to past tobacco use status entries.

**Results:**

An overall current tobacco use prevalence of 27.1% was found during the study period. Tobacco use status was recorded in 96% of visits. Comparison of initial to final visit tobacco use status demonstrates a consistency rate of 75.0% declaring no change in tobacco status in the 4,522 patients with two or more visits. An 8.6% net tobacco use decline was seen for the practice (p value < 0.001).

**Conclusion:**

Self reported tobacco use status as a vital sign embedded within the workflow of an electronic medical record enabled practice was a quantitative tool for determination of tobacco use prevalence and a measuring stick of risk prevention/intervention impact.

## Background

Public health guidelines have long recommended that patient-professed tobacco use status be assessed for every patient at every clinical care visit as a vital sign [1]. In actual clinical practice this recommendation acts as a prompt for tobacco intervention to the medical team [2,3]. Implementation of this recommendation within an electronic medical record has been described in comparison to a paper system with tracking codes [4]. We report on a non-physician mediated means of determining the prevalence of tobacco use and impact of tobacco prevention/treatment efforts in an electronic medical record enabled practice utilizing a defined tobacco vital sign variable.

Electronic medical records have recently been advocated as valuable tools in the protection of patient safety during medical care [5]. There can be a desirable cost benefit ratio related to using an electronic charting system instead of the traditional paper system [6].

Electronic medical records track issues as variables, in this case tobacco use status. Variables permit the analysis of the discreet data points they represent. Structuring these data points with a limitation on degrees of freedom and a practical mutual exclusivity aids in the quantitative analysis of the variable. Collection of each patient's tobacco use status as "current," "former," or "never" at every ambulatory care visit has been advocated [7]. Following this recommendation provides structure to the tobacco use status data points. Including "no entry" to this list of choices provides mutual exclusivity. Functional incorporation of the data collection process into a routine work flow facilitates the collection of discreet data points for comparison. Examples of this in a clinical setting include graphing of fever curves related to patient temperature or following of blood pressure readings over a series of successive readings.

Undertaking paper medical record based analysis usually involves review of a subset of data instead of all the data that could be extracted from the patient records [8]. This process introduces the complication of sample error during the analysis [9]. Groups have attempted to automate this effort through the tracking of tobacco use status in electronic systems such as registration systems. Electronic systems offer the opportunity to overcome sample error by extracting all discreet data entries in a given category with great efficiency. Electronic medical records take this a step further by allowing clinical recommendations to be embedded into a specific clinical workflow. These systems have been reported to contain more data related to observations and patient education than paper records [10].

Successful incorporation of a process into a clinical workflow requires an efficient embedding of the process into the normal workflow of the practice [11]. Vital signs are frequently obtained in the pre-provider portion of patient care visits. This is the period of the visit at which time the nurse or medical technician calls the patient from the practice's reception area and brings the patient to a place for the taking of blood pressure, pulse, height, weight, respiratory rate, temperature, and patient-specific tests such as a finger stick blood sugar in a diabetic patient or a peak flow rate in an asthmatic patient.

We chose to study the characteristics and variability of the non-physician obtained EMR facilitated tobacco vital sign.

## Methods

We present a retrospective cohort study beginning July 15, 2001, and ending May 31, 2003.

### Clinic Setting

UMD Care is the general internal medicine ambulatory care practice of the University Hospital in Newark, New Jersey. The practice is considered a safety net practice within a safety net hospital. The patients of this practice are as a group socioeconomically needy. The practice's staff includes five full-time attending physicians, six part-time resident practice attending physicians, 36 internal medicine residents, and 20 support staff. Both private practice and resident teaching practice is conducted at this site. An electronic medical record, Logician TM (GE Medical Systems Inc., Hillsboro, Oregon), has been in use at the practice since May 1997. All clinical staff utilize the EMR for documentation of every visit.

### Vital Sign Acquisition Process

**Figure 1:**
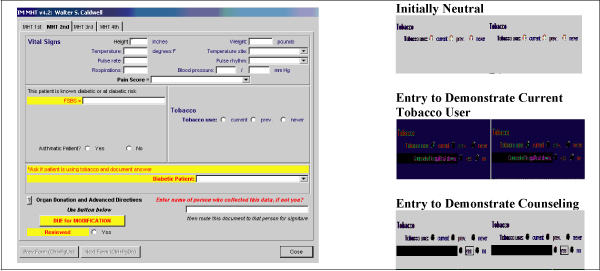


All patients are seen initially by a medical health technician (MHT) who records vital signs. MHTs were instructed to include a patient-professed tobacco use history as a vital sign utilizing a computer screen as shown in the simulated patient record in Figure 1. Documentation of tobacco use is categorized as "current," "previous," or "never." All current tobacco users are counseled to quit by the MHT and a counseling box is checked to document related prevention education. The MHT was blinded to the previously reported tobacco status in order to provide an unbiased assessment (Figure 1). The provider has access to this information during the provider component of the visit. Data were extracted from the EMR database with the approval of our institutional review board.

### Vital Sign Data Extraction

Data collected for each patient included age, sex, race, date of visit, and tobacco status at each visit, and tobacco counseling. Patients' data was de-identified by using patient age at the time of the report, instead of the more specific birth date. Patients were given an integer as identification to provide a verification mechanism to verify the application of the appropriate data to the appropriate patient.

### Tobacco Therapy

A list of all bupropion and nicotine therapy was extracted from the database for the population. All data were de-identified as described above and combined with that of the vital sign data extraction process in order to permit analysis.

### Tobacco Related Counseling

MHTs recorded the provision of their counseling of the patient to "stop smoking" by checking a box on the vital signs form. Physician counseling would be recorded as free text, not a discreet variable, thus is not analyzed in this report.

### Outcomes

Demographic data were extracted based on age at time of report, gender and race for the population. Tobacco use status was then analyzed for compliance with all entries and initial visit tobacco use prevalence. For patients with two or more visits, tobacco use statuses were compared for consistency. Consistency was defined as no change in tobacco status from visit to visit. If there was no change in visit-to-visit tobacco use status, this was inferred to mean the patient's use of tobacco had not changed between visits, while any changes in status between data points permitted characterization of that change. A person initially documented as reporting "current" tobacco use and later being documented as having "previous" tobacco use could be inferred to have achieved cessation at that point in time. Any person initially documented as reporting "never" tobacco use and later being documented as having "current" tobacco use would be inferred to have started tobacco use in the interim period. A person who had achieved cessation but relapsed into tobacco use would have a change from "previous" to "current." First visit and last visit data were then placed in visit aggregates and compared for differences in order to obtain a net tobacco change over the study period.

It has long been recognized that physicians could increase their use of pharmacologic therapies in tobacco cessation [12]. Tobacco therapy was represented by evidence of a prescription for bupropion or a nicotine replacement therapy or evidence of tobacco cessation counseling as documented by the MHT.

### Data analysis

Univariate analysis was applied to study data. Tobacco use prevalence as reported in the Behavioral Risk Factor Surveillance Survey (BRFSS) of the Center for Disease Control and Prevention for the year 2000 was used to compare study data to that of state and nation [13].

## Results

There were 6,771 patients in the data set with 24,824 visits; this provided 24,824 tobacco use status data points for the analysis. Tobacco use status data were present in 96%. Average visits per patient were 3.6 for the study period with a range of one visit per patient to 39 visits per patient. Gender of the population was 59.2% female and 40.8% male. Black and Hispanic patients made up 81.6% of these individuals. Average age of the patients was 51.2 years with a range from 16 years to 98 years (Table 1).

**Table 1:** Demographics

**Age (years)**		**Gender**		**Race**	
Average	51.2	Male	40.78%	Black	59.9%

Range	16 – 98	Female	59.20%	Hispanic	21.8%

		Blank	0.02%	White	4.8%
		
				Other	13.5%

**Figure 2:**
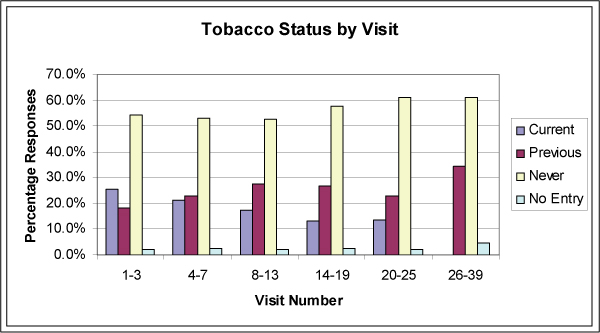
**Tobacco use status aggregates by visit number**.

Evaluation of aggregated visit statuses demonstrates a trend for a decreasing percentage of patient current tobacco use with increasing numbers of visits over the study period (Figure 2). When compared to tobacco prevalence as determined by the BRFSS, the practice's 27.1% current tobacco use rate was outside the confidence interval for New Jersey (19.4 to 22.5), though it is in line with published reports that suggest urban populations are more prone to active tobacco use (Table 2).

**Table 2:** Percent Current Tobacco Use by Initial Tobacco Status

	**Total Current**	**Current Males**	**Current Females**	**18 – 34 Years**	**35 – 49 Years**	**50 – 64 Years**	**> 64 Years**
	% CI n	% CI n	% CI n	% CI n	% CI n	% CI n	% CI n

**UMD Care Study**	**27.1** - 6771	**34.6** - 2761	**22.0** - 4009	**25.4%** - 1037	**34.2%** - 2007	**30.0%** - 2414	**14.3%** - 1311

**New Jersey 2000**	21.0 (19.4–22.5) 783	23.6 (21.0–26.1) 330	18.6 (16.8–20.3) 453	25.4 (22.2–28.5)234	22.9 (20.1–25.6) 313	20.9 (17.5–24.2) 153	10.6 (8.0–13.1) 77

National (USA) 2000*	23.2	24.4	21.2	28.1	26.4	21.1	9.7

CI = Confidence Intervals

N = Number of surveyed individuals in that group

Patients with two or more visits totaled 4,522 individuals. This group's average number of visits was 3.6 with a range of 2 to 39 visits. Evaluation between first and last visit for these patients demonstrated a 75.0 consistency rate in entries (Table 3). The largest variability, 12.4%, was found in those patients for whom the only entries were "never" and "previous" in either order. All other variability made up only 12.6% of the entries.

**Table 3:** Consistency of Data Entries by Patient

**Variability between Medical Health Technician entries**	**Patient Number**	**Patient Percent**
**No tobacco status variability**	3392	75.0%

**Patient with combinations of never and previous only**	559	12.4%

**All other tobacco status changes (current to previous, etc.)**	571	12.6%

Aggregate tobacco use statuses on first visit (Table 4) and on final visit (Table 5) were compared. Current tobacco use decreased from 1,146 patients on the initial visit to 1,047 on the final visit (Table 6). This is a net patient tobacco cessation of 99 patients or 8.6% (P value < 0.001).

Tobacco cessation counseling was present in 91.3% of patient care visits. Tobacco therapies were listed in only 20 charts. Analysis of patient medication lists indicated three bupropion and 17 nicotine replacement prescriptions.

## Conclusion

Tobacco is a major modifiable risk factor for several diseases and conditions [14]. The recorded tobacco use status professed by patients is now an advocated measure of tobacco use prevalence. The character of these data points is not well described for variability and trending, but has become the chief tool for the measurement of quality of care provided by physicians and health care organizations in regard to tobacco intervention. This concern is especially true when related to its documentation by non-physicians during the acquisition of vital signs for routine patient care visits in an ambulatory setting.

The described electronic medical record enhanced process lends itself to the characterization of tobacco use trends in individuals and populations over a series of care visits. Undertaking paper medical record based analysis would often involve review of a subset of data instead of all the data that could be extracted from the patient records, introducing the further complication of sample error in the analysis. This is required due to the labor requirements of manual data capture. Electronic systems offer the opportunity to overcome sample error by extracting all discreet data entries in a given category with great efficiency [15]. Also, they provide a sample set that in this example was the entire population, therefore it was unnecessary to calculate the confidence interval.

Groups have attempted to automate this effort through the tracking of tobacco use status in registration electronic systems [16]. Electronic medical records take this a step further by allowing clinical recommendations to be embedded into a specific clinical workflow [17]. This process in these systems permits the tobacco use status to be linked to other clinical data points forming a tobacco use registry. The cost of the process is negligible. The questions are asked while performing other vital sign related tasks. Benefits obtained using this type of system during the study period have been demonstrated to be maintained well beyond the end of that study [18].

Of the patients present within our study, 96% had a tobacco use status recorded, suggesting that the staff was compliant with addressing the documentation requirements. There was no change in status for 73.2% of patients concerning their first and last visit during the study period despite blinding the MHT to the previous tobacco use history of the individual patients. The proportion of current tobacco use by our population meets prevalence expectations suggested by other published works for similar populations, suggesting compliance with the process by staff. Analysis of patients with variability in these entries shows a predilection for a "Never/Previous" phenomenon. For reasons unclear in the data, a relatively large segment of the patient population was documented as having self-reported tobacco status alternating between "never" and "previous." Whether this is a product of patients minimizing their report of a socially negative behavior or an error related to the documenter's technique for acquiring the data or some other factor, we believe this warrants further investigation.

We did not expect the use of the tobacco use vital sign to demonstrate alteration in physician behavior significantly at this point [19]. Our attempt to identify the interventions provided by physicians to their patients was not easily accomplished. Pharmacotherapy is an advocated treatment process. A review of the medication lists of all patients who had been included in the study demonstrated only 20 medication orders. As prescriptions are printed in the practice from the electronic medical record for all medications it is unlikely any physician is hand-writing these prescriptions. Co-morbidities and contraindications were not screened in this analysis. The socioeconomic nature of the population is likely also a factor, in that patients may not be accepting prescriptions for medications they cannot afford or are not covered by their health plans. Physicians could be projecting a bias that their patients would refuse prescriptions for medications they cannot afford or are not covered by their health plans. Over-the-counter cessation aids may be discussed with the patients but not included in the medication list. Referral to state funded programs [20,21] would be embedded in text and not structured in discreet, trackable variables. Educational sessions have been started for physicians and staff in an attempt to have a discussion on the issue and provide training in tobacco treatment aids as a prelude to further study [22].

A weaknesses of this study was the accuracy of the tobacco status being professed by the patient to the documenting non-physician staff member and the correct subsequent documentation of that data into the record. Consistency of the data and the percentage of current tobacco users are supportive of the correctness of the data. It is possible to attempt to confirm the tobacco use status at each visit using cotinine or carbon monoxide testing, however, neither was incorporated into this study due to concerns about the impact on practice workflow and costs associated with each process. The use of point prevalence tobacco use status should not be the standard by which tobacco use status is used to define true tobacco cessation.

Work continues on this project at this time. The vital sign has been augmented with a length of time from last tobacco use query to go beyond the point prevalence limitation we described. Feedback to providers and staffs related to their interventions and impact has begun as a step toward academic detailing of providers [23]. Expansion of the process to ambulatory care practices of two other medical schools has recently taken place. Tailoring of the process as a component of inpatient initial order entry for all admissions at our University Hospital with primary diagnosis of acute myocardial infarction and congestive heart failure has been approved by the hospital's clinical practice committee of the medical staff. Surveys of patients, non-physician healthcare workers, and physicians are being developed at this time to assess qualitative value and barriers created by the process. Development and study of point of care, decision support tools based on the EMR applied tobacco use vital sign are in the prototype phase of evaluation.

## Competing interests

The authors declare that they have no competing interests.

## References

[B1] Treating Tobacco Use and Dependence. Summary, June 2000.

[B2] Robinson MD, Laurent SL, Little JM (1995). Including smoking status as a new vital sign: it works!. Journal of Family Practice.

[B3] Ahluwalia JS, Gibson CA, Kenney RE, Wallace DD, Resnicow K (1999). Smoking Status as a Vital Sign. Journal of General Internal Medicine.

[B4] Bentz CJ, Davis N, Bayley B (2002). The feasibility of paper-based Tracking Codes and electronic medical record systems to monitor tobacco-use assessment and intervention in an Individual Practice Association (IPA) Model health maintenance organization (HMO). Nicotine & Tobacco Research.

[B5] Bates DW, Cohen M, Leape LL, Overhage JM, Shabot MM, Sheridan T (2001). Reducing the frequency of errors in medicine using information technology. Journal of the American Medical Informatics Association.

[B6] Wang SJ, Middleton B, Prosser LA, Bardon CG, Spurr CD, Carchidi PJ, Kittler AF, Goldszer RC, Fairchild DG, Sussman AJ, Kuperman GJ, Bates DW (2003). A cost-benefit analysis of electronic medical records in primary care. American Journal of Medicine.

[B7] Fiore MC, Jorenby DE, Schensky AE, Smith SS, Bauer RR, Baker TB (1995). Smoking status as the new vital sign: effect on assessment and intervention in patients who smoke. Mayo Clinic Proceedings.

[B8] Norman LA, Hardin PA, Lester E, Stinton S, Vincent EC (1995). Computer-assisted quality improvement in an ambulatory care setting: a follow-up report. Joint Commission Journal on Quality Improvement.

[B9] Lawthers AG (1996). Validity review of performance measures. International Journal for Quality in Health Care.

[B10] Hippisley-Cox J, Pringle M, Cater R, Wynn A, Hammersley V, Coupland C, Hapgood R, Horsfield P, Teasdale S, Johnson C (2003). The electronic patient record in primary care – regression or progression? A cross sectional study. British Medical Journal.

[B11] Briggs B (2002). Electronic medical records: a "work-flow" in progress. Health Data Management.

[B12] Thorndike AN, Rigotti NA, Stafford RS, Singer DE (1998). National patterns in the treatment of smokers by physicians. Journal of the American Medical Association.

[B13] Centers for Disease Control and Prevention (CDC) (2000). Behavioral Risk Factor Surveillance System Survey Data.

[B14] Hecht SS (2003). Tobacco carcinogens, their biomarkers and tobacco-induced cancer. Nature Reviews. Cancer.

[B15] Bates DW, Cohen M, Leape LL, Overhage JM, Shabot MM, Sheridan T (2001). Reducing the frequency of errors in medicine using information technology. Journal of the American Medical Informatics Association.

[B16] McAfee T, Grossman R, Dacey S, McClure J (2002). Capturing tobacco status using an automated billing system: steps toward a tobacco registry. Nicotine & Tobacco Research.

[B17] Ornstein SM, Jenkins RG, MacFarlane L, Glaser A, Snyder K, Gundrum T (1998). Electronic medical records as tools for quality improvement in ambulatory practice: theory and a case study. Topics in Health Information Management.

[B18] Cooley KA, Frame PS, Eberly SW (1999). After the grant runs out. Long-term provider health maintenance compliance using a computer-based tracking system. Archives of Family Medicine.

[B19] Piper ME, Fiore MC, Smith SS, Jorenby DE, Wilson JR, Zehner ME, Baker TB (2003). Use of the vital sign stamp as a systematic screening tool to promote smoking cessation. Mayo Clinic Proceedings.

[B20] Steinberg MB, Delnevo CD, Hrywna M (2002). Update in New Jersey tobacco-dependence treatment. New Jersey Medicine.

[B21] Foulds J, Burke M, Richardson D, Kazimir E (2002). Tobacco dependence treatment services in New Jersey. New Jersey Medicine.

[B22] Lindsay EA, Ockene JK, Hymowitz N, Giffen C, Berger L, Pomrehn P (1994). Physicians and smoking cessation. A survey of office procedures and practices in the Community Intervention Trial for Smoking Cessation. Archives of Family Medicine.

[B23] Hosler AS, Godley K, Rowland DH (2002). An initiative to improve diabetes care standards in healthcare organizations serving minorities. Diabetes Educator.

